# Wolf (*Canis lupus*) Generation Time and Proportion of Current Breeding Females by Age

**DOI:** 10.1371/journal.pone.0156682

**Published:** 2016-06-03

**Authors:** L. David Mech, Shannon M. Barber-Meyer, John Erb

**Affiliations:** 1 U. S. Geological Survey, Northern Prairie Wildlife Research Center, Jamestown, North Dakota, United States of America; 2 Minnesota Department of Natural Resources, Grand Rapids, Minnesota, United States of America; University of Southern Queensland, AUSTRALIA

## Abstract

Information is sparse about aspects of female wolf (*Canis lupus*) breeding in the wild, including age of first reproduction, mean age of primiparity, generation time, and proportion of each age that breeds in any given year. We studied these subjects in 86 wolves (113 captures) in the Superior National Forest (SNF), Minnesota (MN), during 1972–2013 where wolves were legally protected for most of the period, and in 159 harvested wolves from throughout MN wolf range during 2012–2014. Breeding status of SNF wolves were assessed via nipple measurements, and wolves from throughout MN wolf range, by placental scars. In the SNF, proportions of currently breeding females (those breeding in the year sampled) ranged from 19% at age 2 to 80% at age 5, and from throughout wolf range, from 33% at age 2 to 100% at age 7. Excluding pups and yearlings, only 33% to 36% of SNF females and 58% of females from throughout MN wolf range bred in any given year. Generation time for SNF wolves was 4.3 years and for MN wolf range, 4.7 years. These findings will be useful in modeling wolf population dynamics and in wolf genetic and dog-domestication studies.

## Introduction

Although some basics are known about reproduction in female gray wolves (*Canis lupus*) [1], little or no information exists about the mean age of first reproduction, generation time, and the proportion of females that breed in wild populations during any given year. Gravid wild wolves < 1-year old have been documented [2]; a few captive wolves have bred when 10-months old [3], [4]; wild wolves bearing pups at 1-year of age in Belarus have been reported (V. Sidorovich, personal communication); and a wild Yellowstone National Park wolf whelped at 1 year [1]. However, these records are rare. In some areas it is common for wild wolves 2-years old to breed ([5]) whereas other wolves do not breed until 4 or 5 years of age [6], [7]. Ovarian and uterine weights maximized in wolves ≥ 3-years old in Canada’s Northwest Territories [1], suggesting that females there are sexually mature at that age.

Information about proportion of wild female wolves breeding at various ages and in any given year is basic to a more realistic understanding of wolf biotic potential yet no such information is available. It would also be valuable for modeling wolf populations [8] and for an accurate calculation of generation time for wolf genetic research and dog domestication studies, and it would inform studies concerned about possible lack of breeding in small inbred populations such as that on Isle Royale [9]. Mech [10] conducted a preliminary study of breeding status of gray wolves for ages 2–12 and found that some females of each age had failed to breed. However that investigation involved only 23 wolves.

Thus we evaluated the proportion of female wolves of various ages that bred in any given year in a long-extant population in northeastern MN as well as throughout MN wolf range. Our objectives were to determine for female wolves in a given population (1) the variation in ages of first breeding, (2) the proportion that bred in any given year, (3) the mean age of primiparity, and (4) generation time.

## Methods

Our data are from two sources (S1 Dataset): (1) 41 years of live-captured wolves from an intensively studied 2,060-km^2^ area in the east-central Superior National Forest (SNF) of northeastern MN [11], including the wolves that Mech [10] studied, and (2) 3 years of harvested wolves from throughout the entire approximately 70,000 km^2^ MN wolf range [12].

The SNF study was prescribed by the U.S. Fish and Wildlife Service 1978 and 1992 Eastern Timber Wolf Recovery Plans and conducted before, during and after the wolf was Federally listed as endangered or threatened. Data were collected under the following permits: (1) U.S. Fish and Wildlife Service PRT 831774 and TE3886A-0, which allowed “such activities as capture, chemical immobilization, radio-collar, track, salvage, assess and treat health conditions” of gray wolves, and (2) Minnesota Department of Natural Resources (MN DNR) Special Permits 9125, 10317, 11586, 12755, 14003, 15517, and 16856 “for purposes of research, to capture live, transport, and retain gray wolves.” The protocol for capture, anesthetizing, handling and release of study animals was approved by the U.S. Fish and Wildlife Service, Patuxent Wildlife Research Center Institutional Animal Care and Use Committee (IACUC) on September 28, 1990 and the U.S. Geological Survey, Northern Prairie Wildlife Research Center IACUC on December 4, 2015. We followed the guidelines of the American Society of Mammalogists for the Use of Mammals in Research ([13], [14], [15]). The SNF is Federal land, and the study involving live wolves was conducted by Federal biologists on Federal land.

In the SNF study area, wolves feed primarily on white-tailed deer (*Odocoileus virginianus*), moose (*Alces alces*), and, mainly during the ice-free season, beavers (*Castor canadensis*) [16], [17]. The same is true for wolves throughout MN, except that moose are lacking in most of MN wolf range. The SNF wolf population is one of the few studied wolf populations that has never been exterminated [18], although until 1974 its more accessible parts had been exploited [19]. More detailed information about the SNF study area can be found in [11] and about the entire MN wolf range in [20].

In the SNF from 1972 to 2013, primarily during June through October, we live-trapped wolves with modified foot-hold traps, and drugged them intramuscularly with anesthetics via syringe-pole. Several combinations of anesthetics were used during the study: phencyclidine hydrochloride and promazine hydrochloride; ketamine hydrochloride and promazine hydrochloride; and tiletamine hydrochloride, zolazepan hydrochloride, and xylazine (reversed with yohimbine) [1] [21]. The wolves were then weighed, measured, radio-collared, and later aerially located year around and observed, mostly in winter but occasionally other times [21, 11]. We measured length and basal width of nipples with calipers and recorded whether milk could be expelled from them. (Wolves nurse for up to 10 weeks [22], so our study animals could have been nursing or post nursing when captured, and our nipple-classification equation (see below) accounted for that [23, 24].) We monitored respiration rate, rectal temperature, and level of anesthesia, and injected animals with antibiotics in case of any capture or handling injuries. When processing was complete, we left the animals in sternal recumbency to recover free from the stress of human presence, or applied the reversal drugs and observed the animals as they recovered.

We recorded amount of tooth wear, and from 2000 on, we compared the amount of tooth wear to a tooth-wear chart of known-age wolves to assign ages [25]. Wolves captured as pups and later recaptured were known-age. Wolves captured and recaptured before 2000 were generally considered ≥ 1-year old at first capture, and we added the number of years between capture and recapture to assign a known minimum age at later capture. In several of these cases, where first-capture tooth condition indicated an age older than 1, we estimated an original age based on tooth-wear description. We excluded all pups and yearlings from our analyses, for none showed any sign of having bred.

We determined ages and breeding status of SNF female wolves > 2-years old based on 113 captures (86 wolves) (Table 1). Eight wolves (13 captures) were known-age (Table 2). Wolves in 57 captures were aged by comparison with the Gipson et al. [23] tooth-wear chart. Wolves in 43 captures were minimum-aged or produced ambiguous ages in the field from the tooth-wear chart and their ages were adjusted post hoc from descriptions of tooth wear.

**Table 1:** Female wolf breeder types in the Superior National Forest, 1972–2013 based on Mech et al. [24] formula^a^.

				Breeders		
Age	No.	Non-breeders	Former	Current or former^b^	Current	Total (%)
2	34	25	1^c^	4	4	9 (26)
3	28	16	0	5	7	12 (42)
4	18	9	4	3	2	9 (50)
5	11	1	4	1	5	10 (56)
6	3	1	1	0	1	2 (18)
7	7	1	2	1	3	6 (86)
8	3	0	1	0	2	3 (100)
9	4	2	0	2	0	2 (50)
10	0	0	0	0	0	0 (0)
11	1	0	1	0	0	1 (100)
12	1	0	1	0	0	1 (100)
	110	55	15	16	24	55

^a^ In addition, one 3-year-old, one 5-year-old, and one 10-year-old were classed as “non- or former” breeders.

^b^ Wolves with ambiguous classification.

^c^ Probably mis-aged because we have no records of yearlings breeding.

**Table 2:** Information about known-age female wolves ≥ 2-years old in the Superior National Forest, Minnesota captured from 1972 to 2013.

			Nipple	Known	Calculated
Wolf	Capture date	Age	length + width (cm)	breeder status	breeder status^a^
413	6/26/87	3	0.64	non-breeder	non- breeder
413	6/22/88	4	3.3	current	Current
743	8/5/02	4	1.5	probably current^b^	Former
951	9/28/08	4	1.42	probably current^c^	current or former
5139	7/29/76	2	1.55	possibly current^d^	current or former
5176	6/26/76	2	3.47	possibly current^d^	current
5176	9/11/79	5	4.3	Current	former
5176	7/26/81	7	1.27	Current	former
5176	10/12/83	9	1.22	Former	current or former
5176	8/22/85	11	1.13	Former^e^	former
5409	6/1/77	2	-^f^	probably current	-
5415	9/16/78	3	0.65	Unknown	non-breeder
6433	2/12/85	3^g^	-	Immature^g^	-
6439	8/14/86	4	1.27	Unknown	former

^a^Based on Mech et al. [24] formula. “Current or former” indicated that index of ambiguity difference was < 0.20 (see Methods).

^b^Did not localize during denning season when 2 or 3 but did when 4-years old.

^c^Localized when 2 and 4-years old; no data when 3.

^d^ Localized during denning season.

^e^ Bred in 1984.

^f^ No measurements, but nipples “very apparent”.

^g^ 3-yr-old as of April 1985. See also Discussion and [6].

### Interpreting breeding status from nipple size

We determined whether a wolf had bred in its year of capture by applying a classifying formula [24] to the length plus width of the largest nipple and the date captured. The formula was based on biweekly nipple measurements of 4 categories of wolves of known breeding histories: yearlings, adult non-breeders, former breeders, and current breeders [24]. We considered wolves with inconspicuous nipples (small enough that we did not measure them, < 0.2 mm) as non-breeders.

For each wolf with measured nipples, we calculated t-values for each of the 4 above potential categories and then evaluated the two-tailed probabilities of obtaining each t-value in Arc version 1.06 [26]. Hence we tested that the observed nipple size came from the same statistical population as each Mech et al. [24] category [25] (higher probabilities indicated greater likelihood of belonging to that particular breeder category). We assigned each wolf to the breeder category with the highest probability. However, in some cases, the probability of the second-highest-probability (“runner up”) category was very close to that of the assigned category. Therefore, we developed an “index of ambiguity” for each wolf by summing all the resulting probabilities and determining what fraction of this total probability was contributed by each category; contribution fractions summed to 1. Whenever the difference between the contribution fraction from the selected category and the “runner up” category was less than 0.20, we categorized the wolf as potentially in either category (e.g., “current or former” breeder, “adult non- or former” breeder, etc.; Table 1). However, because we excluded yearlings, we lumped any older wolves the formula incorrectly categorized as yearlings due to their small nipples into a non-breeder category that also included adult non-breeders. Thus we compiled our results into 3 categories: non-breeders, former breeders, and current breeders.

In a few cases the formula produced results that contradicted the known status of our study wolves (see Results). In these cases, the formula classed the wolves as “former breeders,” but for our final assessment we classed those females as “current breeders” if they were lactating, if pups were present and they were the only known adult female in the pack, or if there were sample-size or variance problems in the Mech et al. [24] captive-wolf nipple data that resulted in wolves with large nipples being errantly classed as former breeders (see below and [25]).

### Minnesota wolf range data

The data from throughout the MN wolf range were collected by MN DNR authorities from carcasses of legally harvested, hunter/trapper-killed wolves from November 2012 through December 2014 whose reproductive tracts were examined for placental scars [5], and whose ages were estimated from tooth-sectioning by Matson’s Laboratory, LLC., Milltown, Montana, USA [27]. In this sample, successful breeding was equated with the presence of placental scars, whereas in the SNF sample successful breeding was equated with pups surviving to the nursing stage.

### Mean age of primiparity and generation time

We calculated mean age of primiparity as follows: We (1) considered all the 2-year-old breeders (bred at 22 months) as first breeders, because none of our SNF yearlings had measurements consistent with a breeder and because of so little evidence that younger wolves breed, (2) subtracted the percentage of 2-year-old breeders from the percentage of 3-year old breeders and multiplied that percentage times the total number of wolves of that age to determine the number of 3-year-old first-breeders, (3) proceeded similarly with each successive age in which the percent of breeders was higher, (4) multiplied the total first-breeders of each age times that age to yield the number of cohort-years of first-breeders for each age, and (5) divided the total number of cohort-years by the total number of first breeders to yield the mean age of primiparity. This approach assumes that each age cohort had the same first-breeder history as the preceding ones. For example, this approach assumes that when they were 2-years old, the 3-year olds in our sample bred in the same proportion as our 2-year old cohort. For the SNF data, this approach also assumes that the data collected over a long period represent a reasonable approximation of the general trend in primiparity over that period. For the data from throughout MN wolf range, that sample represents the mean age of primiparity during 2012, 2013, and 2014.

We calculated generation time (weighted mean age of breeders) as follows:

$$\bar{x}=\frac{\sum_{i=2}^{n}(w_{i}x_{i})}{\sum_{i=2}^{n}w_{i}}$$ (1)

where $\bar{x}$ = generation time (i.e., the weighted mean age of breeders), w = number of breeders in a particular age category in the year of their capture, and x = age in years for i = 2-years-old through n = maximum age observed.

We used chi-square and t-tests for comparisons and considered all differences significant at alpha = 0.05.

## Results

### Proportion breeding in a given year

We encountered problems with using the formula from Mech et al. [24] to distinguish among non-breeders, former breeders, and current breeders, for many of the biweekly samples in that study were small with large standard deviations and overlapping distributions [25]. Thus relying on the Mech et al. [24] formula alone, we were not able to distinguish 3 non-breeders from former breeders and 16 former breeders from current breeders (Tables 1 and 2).

Nevertheless, we were able to reasonably estimate the proportion of SNF females that produced pups in a given year for specific ages. Assuming that all females we classed as current or “current or former” breeders were in fact current breeders (Table 1), then 36% of 110 SNF females ≥ 2-years old bred in any given year. By using field observations to override the formula’s classifications, and excluding all females where classifications were ambiguous (i.e., “current or former breeders”), then of 98 females, 33% bred in any given year (Table 3). The proportion of the age cohort that bred in any given year increased by age from 19 to 80% for 2-5-year-olds (n = 81) and then declined to an average of 44% for those 6-12-years old (n = 18) (Table 3). However, the difference between the proportion of SNF 2-5-year-old and 6-12-year-old wolves classified as breeders in their capture year as per Table 3 was not significant (*P* = 0.21) Several of the SNF wolves classified as non-breeders showed evidence of breeding in earlier or later years (Table 4).

**Table 3:** Female wolves in the Superior National Forest, 1972–2013, that bred or did not breed in a given year based on the Mech et al. [24] formula and/or our adjustments (8 wolves)^a^ based on field studies. Fifteen wolves with ambiguous classification and no additional field data (Table 1) were excluded.

		Did not breed in a given year			
Age	No.	Non-breeders	Former breeders	Total	Bred in a given year (%)
2	31	25	0	25	6 (19)
3	23	15	0	15	8 (35)
4	16	9	4	13	3 (19)
5	10	1	1	2	8 (80)
6	3	1	1	2	1 (33)
7	7	1	2	3	4(57)
8	3	0	1	1	2 (67)
9	2	2	0	2	0 (0)
10	1	0	1	1	(0)
11	1	0	1	1	0 (0)
12	1	0	1	1	0 (0)
	98	54	12	66	32

^a^ Eight wolves classed in Table 1 as former or current-or-former breeders were adjusted to current breeders because pups were present or nipples were being used. In one of these cases, the wolf was lactating so possibly pseudopregnant. One non-breeder was a known former breeder, and one “non- or former” breeder was a former breeder.

**Table 4:** Details about breeding status of female wolves ≥ 2-years old in the Superior National Forest that did not breed in their year of capture and for which past or future reproductive information is available. No additional information was available for 17 more wolves that did not breed in their year of capture.

				Next year			
			Age	With		
Wolf	Date	Age	Method	mate?	Pups?	Remarks
413	6/26/87	3	Known	Yes	≥ 6	-
845	6/7/02	3	Estimated^a^	Yes	≥ 4	-
875	8/18/01	2	Estimated^b^	-	-	Dispersed
887	8/15/02	9	Estimated^a^	Yes	Yes	Bred in 2003, 2004
897	6/21/03	2	Estimated^b^	-	-	Bred in 2008
911	7/14/03	2	Estimated^b^	Possibly^c^	Possibly^d^	Bred in 2008
913	7/22/03	7	Estimated^b^	Possibly^c^	Possibly^d^	-
935	8/23/04	2	Estimated^b^	-	-	Dispersed
937	9/1/04	2	Estimated^b^	Yes	Possibly^d^	-
955	6/24/05	2	Estimated^b^	Possibly^c^	Possibly^d^	-
963	7/3/05	2	Estimated^b^	Possibly^c^	No	-
997	7/29/06	2	Estimated^b^	Possibly^c^	Possibly^d^	-
5415	9/16/78	3	Known	Yes	Yes	-
5935	5/21/80	2	Estimated^a^	-	No	Inconspicuous nipples when 2 and 3-years-old
6027	12/30/86	7	Estimated^a^	-	-	Bred in 1981, 1982, 1984
6113	8/26/82	2	Estimated^a^	Possibly	Possibly	-
6119	8/13/82	2	Estimated^a^	Yes	-	Bred in 1985, 1988
6494	6/30/83	3	Estimated^a^	Yes	Yes	-
7087	8/2/09	2	Estimated^b^	Possibly	No	With pack since 2009; bred at 4-years old

^a^ Based on subjective assessment of all available information including tooth descriptions but not comparison with tooth-wear chart[23].

^b^ Based on tooth-wear chart [23].

^c^ With pack.

^d^ Localized in spring and summer

Data from 159 harvested female wolves ≥ 2-years old were available from throughout MN wolf range. Of those, 58% had bred in the previous year (Table 5). Similar to the SNF findings, the proportion of the age cohort that bred in any given year increased by age from 33 to 69% for 2-5-year olds. The difference between the number of 2-5-year-old (n = 124) and 6-11-year-old (n = 35) wolves from throughout MN wolf range that had not bred (49% vs 17%) was significant (*P* = < 0.01; Yates Chi^2^ = 10.22; d.f. = 1). Mean number of placental scars in wolves from throughout MN wolf range (Table 5) did not maximize until females were 6-years old, except for one 10-year-old wolf, and the difference between the mean number of placental scars in 2-5-year-old wolves (5.95) and those ≥ 6 (6.86) was marginally significant (*P* = 0.577; t = 1.92; d.f. = 90).

**Table 5:** Proportion of wolves harvested throughout Minnesota wolf range November-January 2012/2013, 2013/2014 and 2014/2015 that had bred the previous spring based on placental scars.

		Breeders		Mean scars per
Age^a^	No.	No.	%	parous wolf
2^b^	45	15	33	5.0
3	37	17	46	5.1
4	26	20	77	6.8
5	16	11	69	7.2
6	14	11	79	7.6
7	4	4	100	7.0
8	8	8	100	6.5
9	6	5	83	5.6
10	2	1	50	8.0
11	1	0	0	-
	159	92	58	

^a^All ages are 1–7 months older than those of the same listed age in the SNF sample because these wolves were sampled that many months later in the year.

^b^ 94 female wolves were estimated to be 1-year old, and of those, 10 (10.6%) had placental scars. Because there are few records of free-ranging 1-year-old wolves breeding, we assumed these 10 were miss-aged, so we eliminated this cohort and results from our analysis (but see Introduction).

The proportion of ≥ 2-year-old female SNF wolves that bred in any given year (33–36%) was significantly less than the corresponding proportion (58%) of wolves from throughout MN range (for Table 3 current breeders,; *P* < 0.001; Pearson’s Chi^2^ = 15.43; d.f. = 1; for Table 1 current plus “current or former” breeders, *P* < 0.001; Pearson’s Chi^2^ = 12.02; d.f. = 1 which assumes that all 16 SNF “current or former” were actually all current breeders).

### Primiparity and generation time

The mean estimated age of primiparity for the SNF was 3.0 and for the full MN wolf- range sample was 2.9 years (Tables 6 and 7). Generation time was 4.3 for the SNF wolves and 4.7 for the full MN wolf range sample.

**Table 6:** Deriving mean age of primiparity from Superior National Forest wolf breeder data in Table 3.

		Breeders^a^		First Breeders		Total
Age	N	No.	%	%	No.	Cohort Years^b^
2	31	9	29	29	9.0	18.0
3	23	12	52	23	5.5	16.5
4	16	9	56	4	0.6	2.4
5	10	10	100	44	4.4	22.0
6	3	2	-	-	-	-
7	7	7	-	-	-	-
8	3	3	-	-	-	-
9	2	2	-	-	-	-
10	1	1	-	-	-	-
11	1	1	-	-	-	-
12	1	1	-	-	-	-
Total	98^c^	57			19.5^d^	58.9^d^

^a^ Includes 12 former breeders, 32 current breeders, and 13 “current or former” breeders.

^b^ Numbers of first breeders times age.

^c^ Includes 54 non-breeders plus all current and former breeders (Table 3).

^d^ Mean age of primiparity (58.9/19.5) = 3.0 years.

**Table 7:** Deriving mean age of primiparity from wolf breeder data from throughout Minnesota wolf range (Table 5).

				First		Total
		Breeders		Breeders		cohort
Age	N	No.	%	%	No.	Years^a^
2	45	15	33	33	15.0	30.0
3	37	17	46	13	4.8	14.4
4	26	20	77	31	8.1	32.4
5	16	11	69	0	0.0	0
6	14	11	79	2	0.3	1.8
7	4	4	100	21	0.8	5.6
8	8	8	100	-	-	-
9	6	5	83	-	-	-
10	2	1	50	-	-	-
11	1	0	0	-	-	-
Total	159	92	-	100	29.0^b^	84.2^b^

^a^ Number of first breeders times age.

^b^ Mean age of primiparity (84.2/29.0) = 2.9 years.

## Discussion

In the SNF, where wolves had never been exterminated and had been legally protected since 1974, proportions of females breeding in the year sampled ranged from 19% at age 2 to 80% at age 5, and from throughout MN wolf range, from 33% at age 2 to 100% at age 7. Excluding pups and yearlings, only 33% to 36% of SNF females and 58% of females from throughout MN range bred in any given year. Generation time for SNF wolves was 4.3 years and for wolves from throughout wolf range, 4.7 years. Wolves from throughout MN wolf range included subpopulations that had colonized since 1990. Our proportions of females breeding in any given year are considerably less than the 66% and 96% “reproductively active wolves in an Alaska wolf population with low and high-density prey bases, respectively [28]. However the Alaska figures were based on whether wolves were in proestrus, estrus, or gravid. Thus they are not strictly comparable to ours. They do, however, raise the possibility that in wolves there can be much reproductive failure between proestrus and birth, including fetal resorption [29]. As wolf populations recover in more areas of the world [30] and more government agencies begin managing wolves and modeling their populations [31], [8], our findings will help add precision to reproductive data inputs.

Our findings about age of reproductive maturity differ from those for the Yellowstone National Park wolf population. Although no information is available about proportion of each Yellowstone age class that bred in any given year, mean age of primiparity there was 2.7 years compared with 2.9 and 3.0 for our samples. Both the Yellowstone and the SNF primiparity calculations were based on pooling multi-year data. Our estimates are based on the assumption that, on average, each year the proportions of each age that had bred is greater than the proportion of the previous age that had bred, up to 5-years old in the SNF and 7-years old for the larger MN wolf population. Although for any given year, this assumption might not be valid, on average it should be. Because our samples are greatly skewed to younger wolves, any misrepresentation in the older samples will affect the estimate of mean age of primiparity only minimally.

The mean litter size in Yellowstone peaked for 4-year-old wolves [32] compared with 6-years old for MN wolves. Litter sizes in Yellowstone were based on pups observed, whereas MN litter sizes were based on placental scars, so the 2 data sets are not strictly comparable. However, in general, trends in average litter sizes for each age should be more validly comparable between the 2 areas because, within each sample, aging methods were consistent, even though they were different between samples.

The differences in ages of primiparity between YNP and SNF wolves parallel differences in age of maximum body mass between the 2 populations. Yellowstone female wolf body mass peaks at 2.75 years [32], whereas for SNF females it peaks at about 6.00 years [10]. This difference in weight and reproductive performance could be attributable to either genetics (wolves reintroduced into Yellowstone originated in western Canada), degree of population maturity, or both. The Yellowstone population originated from a reintroduction in 1995 and 1996 and was released into an area with a prey population of around 16,000 elk (*Cervus elaphus*), so it sustained maximum nutrition [33]. Conversely, the SNF population has existed for millennia, and much of the population from throughout MN wolf range had existed for as long as 40 years [12].

Our generation times (4.3 and 4.7 years) generally accorded with those used with YNP [34] and with Isle Royale [35] wolf modeling, 4.16 and 4.00 years respectively. However, calculations in several studies of how long ago dogs were domesticated from wolves have assumed 3.0 years as wolf generation time (summarized in [36]). These different wolf generation times could have profound effects on the results and conclusions of the latter studies.

We had some problems using the Mech et al. [24] formula and data to distinguish among the various breeding categories, mostly between former and current breeders, based on our known breeding histories and on the probabilities derived from the formula. The problems arose because (1) some biweekly captive samples were small with wide confidence intervals, and (2) wild wolves are much more subject to pup loss.

Of the 55 non-breeding SNF wolves (excluding yearlings), 45 had inconspicuous nipples, including 20 estimated at 2-years old, 12 at 3, 8 at 4, 1 at 5, 1 at 6, 1 at 7, and 2 at 9. Most of their ages were estimated from a tooth-wear chart [23] and could be inaccurate, with older ages over or under-estimated by up to 5 years [23]. Thus our “older” wolves with inconspicuous nipples could have been younger non-breeders. Alternatively, they could have been former breeders whose nipples regressed as with Wolf 6027, which had inconspicuous nipples when at least 7-years old but bred when at least 2, 3, and 5 years old.

Of our known-age SNF wolves, some or all of our three 2-year-olds might have bred during their capture year; of our three 3-year olds, the breeding status of 1 wolf in year of capture was unknown, and 2 had never bred, but 1 did when 4-years old; most of our 4-year olds (3 of 4) bred at that age; a 5 and a 7-year old bred at those ages; and a 9-year old and 11-year old had bred formerly (Table 2). The information based on these known-age wolves and their known breeding histories is consistent with our findings from those based on the nipple measurements of our entire SNF sample and of those from the larger MN population.

Despite the above shortcomings, we could distinguish wolves that did not breed during the year of capture from wolves that were current breeders, thus allowing us to determine for any given year the proportion of females that bred that year. A lower proportion of SNF female wolves bred in a given year at most ages than those from throughout MN wolf range. Three important differences from the SNF data characterized the data from throughout MN wolf range: (1) method of age estimation, (2) method of assessing breeding status, and (3) history of the population and degree of legal protection. The specimens from the larger MN population were aged by tooth sectioning, whereas the ages of the SNF specimens were based on tooth wear. Tooth sectioning tends to be accurate within a year except for older wolves [23]. However, age inaccuracies would tend to be random so probably would not explain the lower rate of bred females in the SNF. In addition, the proportion of both samples of 2-5-years old was almost the same, 81.6 and 80.0, providing some confidence in the aging techniques. Assessment of breeding status based on placental scars would tend to be more accurate than that based on nipple development, except for assessing pre-breeders [37].

The difference in methods of assessing breeding status might explain the finding of lower breeding rate in the SNF, given the difficulty in the latter sample of distinguishing among breeder types. Still, even assuming all the wolves classed as “current or former” breeders were actually current breeders, the proportion of SNF breeders was significantly less than the proportion in the larger MN wolf population. The difference could also be due to the fact that placental scars only indicate that the wolf was gravid, whereas nipple characteristics depend on the animal having whelped and nursed.

Another possibility is the different histories and legal protections of the 2 samples. The SNF population had never been extirpated and had been legally protected for most of the sample period. Thus it had long before reached its saturation equilibrium with its prey so was in minimal nutritional state for survival and reproduction. Conversely much of the wolf-range population had recolonized, gradually expanded its range over the previous 4 decades and was subject to varying degrees of illegal harvest, government depredation control, and for the 3 years of sampling, public harvest. Thus it might not have been as saturated or stable as the SNF population. Other areas from which similar data are available came from populations subjected to harvest or control, and, like the wolf-range data, all show a higher proportion of females breeding than our SNF sample (Table 8).

**Table 8:** Percentage of female wolves excluding pups and yearlings that bred in various populations.

Location	No.	% bred	Reference
Alaska	183	32–41% of 2-year olds	[5]
Alaska	-	90% of ≥ of 3-year olds	[5]
Ontario	17	59% of “adults”	[38]
Alaska	-	71% of ≥ 2-year olds	[39]
Alaska	15	67% of ≥ 2-year olds	[40]
Northwest Territories	97	57% of parous wolves	[41]
Superior National Forest, Minnesota	102	36–40% of ≥ 2-year olds	This study
Minnesota wolf range	122	57% of ≥ 2-year olds	This study

In any case we can conclude that in our mature, long-extant SNF wolf population as well as with wolves in general, a high proportion of younger female wolves do not breed, and that peak reproduction for wolves in general may not be reached until about 5-7-years old, except in newly colonizing populations. That some SNF female wolves 3-4-years old remained reproductively immature is supported by histology showing that they lack c*orpora lutea*, c*orpora albicantia*, and developing follicles [6].

Because of the prolonged period of reproductive maturation in SNF wolves, and because younger wolves constitute a high proportion of the population (Tables 1 and 5; [42] only about one third of the females ≥2-years old breed in any given year. Because 80% of the SNF non-pup population is < 5-years old [42] the number of wolves that breed in that population for more than a few years is probably low.

In assessing wolf breeding condition based on nipple length or even lactation, one must also consider the phenomenon of pseudopregnancy and premature reproductive activity. Pseudopregnancy occurs when a female shows physical or physiological evidence of pregnancy but is not pregnant. Pseudopregnancy has not been reported in wild wolves but is known in captive wolves. Seal et al. [43] described a captive, barren female wolf whose endocrine profile was about the same as those of concurrently pregnant wolves, although those authors did not label the finding as pseudopregnancy. Jochle [44], indicated that pseudopregnancy is obligatory in all non-pregnant canids, including wolves. However, wolves can also show nipple development and other signs of breeding even though they had neither bred nor were pseudopregnant. Two SNF wolves showed such signs although their ovaries were immature [6]. Thus conceivably females that we classed as current or former breeders could actually have been non-breeders. If that is the case, then the proportion of SNF wolves in our sample that bred each year could have been even lower than our data show.

## Supporting Information

S1 Dataset Excel file of Superior National Forest female wolf capture data (Sheet 1) and summary of data from hunter-killed female wolves from throughout Minnesota wolf range (Sheet 2).
(XLSX)
